# Chemiluminescence response induced by mesenteric ischaemia/reperfusion: effect of antioxidative compounds *ex vivo*

**DOI:** 10.2478/v10102-010-0021-3

**Published:** 2010-09

**Authors:** Viera Nosál'ová, Ružena Sotníková, Katarína Drábiková, Silvia Fialová, Daniela Košťálová, Silvia Banášová, Jana Navarová

**Affiliations:** 1 Institute of Experimental Pharmacology & Toxicology, Slovak Academy of Sciences, Bratislava, Slovak Republic; 2 Department of Pharmacognosy and Botany, Faculty of Pharmacy, Comenius University, Bratislava, Slovak Republic

**Keywords:** ischaemia/reperfusion, chemiluminescence, arbutin, stobadine, SMe1EC2

## Abstract

Ischaemia and reperfusion (I/R) play an important role in human pathophysiology as they occur in many clinical conditions and are associated with high morbidity and mortality. Interruption of blood supply rapidly damages metabolically active tissues. Restoration of blood flow after a period of ischaemia may further worsen cell injury due to an increased formation of free radicals. The aim of our work was to assess macroscopically the extent of intestinal pathological changes caused by mesenteric I/R, and to study free radical production by luminol enhanced chemiluminescence (CL) of ileal samples. In further experiments, the antioxidative activity of the drugs tested was evaluated spectrophotometrically by the use of the DPPH radical. We studied the potential protective *ex vivo* effect of the plant origin compound arbutin as well as of the pyridoindole stobadine and its derivative SMe1EC2. I/R induced pronounced haemorrhagic intestinal injury accompanied by increase of myeloperoxidase (MPO) and N-acetyl-β-D-glucosaminidase (NAGA) activity. Compared to sham operated (control) rats, there was only a slight increase of CL response after I/R, probably in association with neutrophil increase, indicated by enhanced MPO activity. All compounds significantly reduced the peak values of CL responses of the ileal samples *ex vivo*, thus reducing the I/R induced increase of free radical production. The antioxidants studied showed a similar inhibitory effect on the CL response influenced by mesenteric I/R. If proved *in vivo*, these compounds would represent potentially useful therapeutic antioxidants.

## Introduction

Ischaemia/reperfusion injury of the intestine is a significant problem in situations such as abdominal aortic aneurysm surgery, small bowel transplantation, cardiopulmonary bypass, and strangulated hernias. It can also occur as a consequence of collapse of systemic circulation, as in hypovolemic and septic shock. Restoration of blood supply after a critical period of ischaemia results in parenchymal injury and dysfunction of the organ. Certain tissues, such as intestinal mucosa, are especially sensitive to I/R induced injury, which is responsible for an increase of vascular and mucosal permeability, accumulation of neutrophils in the injured mucosa and lesion formation.

Reactive oxygen species (ROS) are supposed to contribute significantly to tissue damage. Thus compounds with antiradical activity may prove beneficial as they help to reduce or even to prevent this damage. Plant derived phenolics represent a good source of natural antioxidants. Some of them have already proved effective against various tissue injuries. Their pharmacological actions stem mainly from their free radical scavenging and metal chelating properties, as well as from their effects on cell signalling pathways and on gene expression (Soobrattee *et al*., 2005). Arbutin was chosen in our experiments as their representative. Among other compounds, the pyridoindole stobadine and its derivative SMe1EC2 were found to have scavenging and antioxidant activities (Horáková *et al*., 1994). The aim of our work was to describe the I/R-induced mesenteric injury and to determine the extent of intestinal damage by assessing the activity of the lysosomal enzyme NAGA as well as the activity of MPO, as an indicator of neutrophil infiltration. Further, the possible protective *ex vivo* effect of some antioxidants on luminol enhanced chemiluminescence was studied on using this model of mesenteric I/R.

## Methods

### Ischaemia/reperfusion

Experiments were approved by the State Veterinary and Food Administration. Male Wistar rats weighing 220–280 g were used. The animals were kept under controlled light and temperature conditions and fed a standard pelleted rodent diet, with access to tap water and diet *ad libitum*. They were acclimatised for one week before experiments. A model of mesenteric I/R was used in anaesthesised rats (Nosál'ová *et al*., 2005), with sham operated animals as controls. Ischaemia was induced by occluding the superior mesenteric artery (SMA) for 60 min, reperfusion lasting 30 minutes started by removing the clamp. The rats were sacrificed by exsanguination and intestinal samples were taken. The extent of intestinal damage caused by I/R was assessed macroscopically by adding the length of all segments (in cm) with signs of injury and the sum expressed as percentage of the whole intestinal length.

### Activity of myeloperoxidase (MPO)

Activity of MPO, a measure of leukocyte infiltration, was determined in the scraped intestinal mucosa by the modified method of Bradley *et al*. (1982). MPO activity was assessed spectrophotometrically by determining the decomposition of hydrogene peroxide using o-dianisidine as hydrogen donor.

### N-acetyl-β-D-glucosaminidase (NAGA) activity

Activity of the lysosomal enzyme NAGA was determined according to the slightly modified method of Barrett and Heath (1977).

### Chemiluminiscence (CL) assay

Free radical production was assessed by luminol enhanced CL of the ileal samples (Millar *et al*., 1996, Nosál'ová *et al*., 2007). Cross-sectional strips of approximately 50–70 mg wet weight were dissected from the ileum. Samples were placed for 60 min into preoxygenated (95% O_2_ and 5% CO_2_) physiological saline solution (PSS) composed of (in mmol/l): NaCl 122, KCl 5.9, NaHCO_3_ 15, glucose 10, MgCl_2_ 1.25, CaCl_2_ 1.25, then transferred into a cuvette containing 920 µl PSS and 80 µl luminol (final concentration 400 µmol/l). The response was measured and recorded in a lumi-aggregometer. The wet weight of samples was recorded at the end of experiment. The peak values and area under the curve were calculated. Since peak values correlated closely with the area under the curve, they were used in further experiments.

### Testing DPPH (1,1-diphenyl-2-picrylhydrazyl)radical scavenging activity of stobadine, SMe1EC2 and arbutin

The antioxidative activity was determined with the DPPH radical using the spectrophotometric method (von Gadow *et al*., 1997) modified by Buřičová and Réblová (2008). The DPPH radical dissolved in methanol in the concentration of 0.05 mg/ml (100 µl) was added to 50 µl of various concentrations of the drugs tested. The experiments were done at room temperature. The decrease in absorbance at 500 nm was measured after 60 min using a spectrophotometer (Multiscanreader RC, Labsystems, Finland). The radical-scavenging activities of drugs were then expressed as percentage of DPPH radical-scavenging activity using the formula: percentage of DPPH radical-scavenging activity = 100 × [(A_blank_ – A_sample_)/A_blank_]. Analytical grade methanol was used to zero the spectrophotometer. Deionised water (50 µl) and DPPH in methanol (100 µl) was used asa blank. All determinations were performed in triplicates.

The effect of the following compounds on CL was studied *ex vivo*: stobadine and its derivative SMe1EC2, and the plant origin compound arbutin in the concentration of 500 µmol/l. For comparison, also other plant origin compounds as well as some other antioxidants were studied. Intestinal samples taken from the rats exposed to 60-min ischaemia followed by 30 min of reperfusion were incubated with each drug individually for 30 min and then the CL response of the samples was recorded. Intestinal samples taken from sham-operated animals were also evaluated. Intestinal damage induced by I/R was described as the percentage damage of the whole intestinal length.

### Statistical analysis

The values are expressed as mean ± SEM, ANOVA (followed by Tukey's test or Student's t-test as appropriate) was used for statistical analysis, *p*<0.05 was considered significant. pD_2_ values – anegative logarithm of EC_50,_ were calculated from the concentration-response curves.

### Drugs

Thiopental, methanol (Microchem, Slovakia), DPPH, luminol, catalase, superoxide dismuthase, TEMPOL, arbutin and rosmarinic acid (Sigma Aldrich, Germany). Plant origin compounds – mentha villosa extract and curcumine were prepared at the Department of Pharmacognosy and Botany, Faculty of Pharmacy, Comenius University, Bratislava. Stobadine and its derivative SMe1EC2 were synthetised at the Institute of Experimental Pharmacology and Toxicology SASc.

## Results

### Antioxidative activity of drugs studied by DPPH test

**Figure 1:**
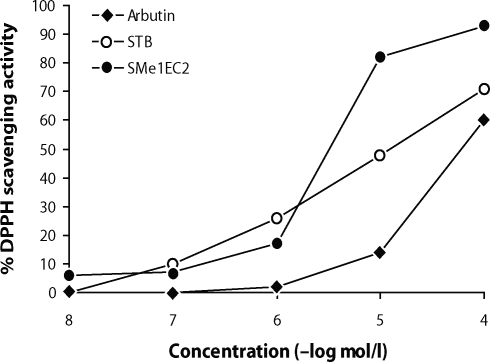
The DPPH radical scavenging effect of arbutin, stobadine (STB) and its derivative SMe1EC2 expressed in percentage. Time 60 min, concentration of the compounds 5×10^–8^ – 5×10^–4^ mol/l.

To select a drug suitable for further experiments, antioxidative activity of each drug used in our experiments was evaluated by the DPPH test. When DPPH (a stable free radical with dark violet colour absorbed at 517 nm) reacts with an antioxidant compound which can donate hydrogen, it is reduced and changes its colour to yellow. As shown in Figure 1, SMe1EC2 exhibited higher DPPH radical-scavenging activity than did stobadine. The scavenging activity of SMe1EC2 was 93% at 5×10^–4^ mol/l and pD_2_ value was 5.67, whereas the scavenging activity of stobadine at the same concentration was 71% and pD_2_ value 4.98. Arbutin exerted a lower effect than did the two previous compounds. Its maximal DPPH scavenging activity was 60% and the pD_2_ value 4.24.

### Ischaemia/reperfusion-induced intestinal damage

Following mesenteric I/R, pronounced intestinal injury of the small intestine was observed, covering 33.5 ± 2.8% of the whole intestine. Injuries of various intensities were found, ranging from hyperaemia to severe haemorrhagic necrosis and bleeding into the lumen. Maximal changes occurred in the terminal ileum. The injury was not distributed homogenously alongside the small intestine: segments with very pronounced injury but also sites without any evident damage were found. Thus samples from both injured and intact sites were taken in some experiments to be used in further examinations.

MPO activity, a measure of leukocyte infiltration, significantly increased by I/R in the mucosa of both noninjured and haemorrhagically injured parts of the small intestine. Similarly, NAGA activity increase was observed after I/R without a difference between samples from injured and noninjured parts of the small intestine (Table 1).

**Table 1:** Changes of intestinal myeloperoxidase and NAGA activity induced by mesenteric ischaemia/reperfusion in rats.

	small intestine	
	
Group	intact sample	haemorrhagic sample
**MPO**		
Control	127.29 ± 10.33	155.00 ± 38.49
I/R	282.17 ± 24.68 ^*^	250.18 ± 26.51^*^
**NAGA**		
Control	8.81 ± 0.55	8.22 ± 0.47
I/R	10.09 ± 0.51^*^	10.76 ± 0.25^*^

*p<0.05 Control versus I/R, data are means ± SEM of n=5–7 for each group MPO – myeloperoxidase, NAGA-N-acetyl-β-D-glucosaminidase

### Effect of drugs on CL changes induced by I/R

Compared to sham operated (control) rats, the CL response increased slightly after I/R, probably in association with neutrophil increase, as indicated by the enhanced MPO activity. There was a difference of CL responses taken from the same intestine, depending on the presence or absence of pathologic changes, mainly haemorrhage. Contrary to expectation, higher values occurred in relatively intact tissue samples. The effects of various plant origin compounds are summarised in Table 2. Based on these results, arbutin was chosen for further experiments.

**Table 2:** Intestinal chemiluminescence in mesenteric ischaemia/reperfusion: effect of various plant origin compounds.

Group	CL peaks ileum intact (cm/100mg)	Cl peaks ileum haemorrhagic (cm/100mg)
Control	16.97 ± 9.63	6.41 ± 1.23
I/R	17.77 ± 3.98	11.12 ± 2.23^*^
I/R AR	4.27 ± 0.89^*^	1.77 ± 0.33^*^
I/R MV	4.67 ± 0.88^*^	3.97 ± 1.62^*^
I/R RA	9.33 ± 7.15	5.24 ± 4.47^*^
I/R CU	16.33 ± 6.91	8.29 ± 2.39

Data are means±SEM, n=5–6 for each group,

*p<0.05 versus I/R.

CL peaks are expressed as cm/100 mg ww from almost intact and/or haemorrhagic ileal samples. I – ischaemia, R – reperfusion, AR – arbutin, MV – mentha villosa extract, RA-rosmarinic acid, CU – curcumine (compounds+I/R in all cases).

The compounds studied *ex vivo,* i.e. arbutin, stobadine and SMe1EC2, reduced significantly the peak values of CL responses of the ileal samples. Moreover, various other antioxidants also reduced the CL response (Table 3). Each compound tested has its own control (initial) value, calculated percentage of control and percentage of inhibition. The most effective compound was the derivative of stobadine, SMe1EC2, with 77% inhibition.

**Table 3:** Intestinal chemiluminescence changes induced by mesenteric ischaemia/reperfusion.

	treatment (–)	treatment (+)	% control	% inhibition
I/R STO	4.28 ± 0.82	1.95 ± 0.27	45.56	54.44
I/R SME	4.37 ± 1.16	1.00 ± 0.31^*^	22.88	77.12
I/R CAT	4.11 ± 0.63	2.19 ± 0.51	53.28	46.72
I/R SOD	4.51 ± 0.69	4.35 ± 1.29	96.45	3.55
I/R TEMPOL	4.36 ± 1.99	1.81 ± 0.07^*^	41.51	58.49

Ileal samples data are mean±SEM, n=5–6, with (+) or without (–) treatment. CL peak values are expressed as cm/100 mg ww, in percent of control (initial) values and percent inhibition. I-ischaemia R-reperfusion, STO-stobadine, SME-stobadine derivative SMe1EC2, AR-arbutin, CAT-catalase, SOD-superoxide dismutase.

*p<0.05 versus treatment (–).

## Discussion

In various experiments, intestinal biopsy specimens from I/R rats produced higher CL than those of sham operated rats, due to ROS production during reperfusion. Similar results were found by Henry *et al*. (1990) in rat hearts subjected to myocardial I/R injury. However, Hamar *et al*. (2003) studying intestinal I/R did not observe an increase in spontaneous ROS production following I/R. Increased MPO activity accompanies mesenteric I/R (Nosál'ová *et al*., 2008) and it is taken as ameasure of neutrophil infiltration. Lojek *et al*. (1997) observed a similar elevation in MPO activity in a model of I/R, in which increased MPO activity was already evident at the end of the ischaemic period, and reached its maximum 3 h after onset of reperfusion. It was shown that neutrophil count increased not only in the intestinal tissue but also in the whole blood after reperfusion (Nosál'ová *et al*., 2008). Activated neutrophils form and liberate ROS both intra-and extracellularly: intracellular oxidant production has a regulatory role in elimination of pathogens, while extracellular ROS generation results in inflammatory tissue damage (Jančinová *et al*., 2006). By using luminol (to record intracellular light emission), a potentiation of CL was found in intestinal tissue samples after *in vivo* administration of different compounds. Increased CL values indicate that further migration of neutrophils into the injured tissue might be facilitated. This is in contrast to our *ex vivo* experiments, where evident inhibition of CL peaks was found.

ROS are supposed to contribute significantly to tissue damage, thus compounds with antiradical activity may prove beneficial as they help to reduce tissue injury. The pharmacological action of phenolic antioxidants stem mainly from their free radical scavenging and metal chelating properties as well as from the effects on cell signalling pathways and on gene expression (Soobrattee *et al*., 2005). The plant origin compound studied in our experiment – arbutin, was found to reduce luminol enhanced CL, suggesting inhibition of ROS increase after I/R. Arbutin, a glycosylated benzoquinone, was found to possess antiseptic and diuretic properties, the ability to decrease allergic inflammatory reaction and the capacity to potentiate antiinflammatory effects of indomethacin and corticoids. It interacts with some processes involved in activation of neutrophil oxidative burst (Jančinová *et al*., 2007).

Experiments with the synthetis compound stobadine revealed a broad spectrum of its activities, including antioxidant and scavenging activity, corroborated by the present results. The beneficial role of stobadine was documented in I/R injury to different vascular beds, e.g. coronary, gastric and cerebral (Nosál'ová *et al*., 2007).

In conclusion, I/R induced a profound injury in the mesenteric region, expressed mainly as increased vascular permeability with protein leakage and subsequent haemorrhage, accompanied by an increase of MPO and CL as a result of increased ROS formation. The *ex vivo* obtained results showed a comparable inhibitory effect of the compounds studied on the peak CL response of intestinal samples influenced by mesenteric I/R. If proved *in vivo*, these compounds would represent a potential source of therapeutically useful antioxidants.
